# A Surgical Conundrum: Addressing a Large Nasal Defect Following Mohs Micrographic Surgery

**DOI:** 10.7759/cureus.68304

**Published:** 2024-08-31

**Authors:** Alexis Arza, Gabriella Vasile, Christopher Buckley

**Affiliations:** 1 Dermatology, Drexel University College of Medicine, Philadelphia, USA; 2 Mohs Micrographic Surgery, Réforme Dermatology, Charleston, USA; 3 Mohs Micrographic Surgery, Philadelphia College of Osteopathic Medicine, Philadelphia, USA

**Keywords:** facial plastic surgery, dermatology, nasal defect, basal cell carcinoma, mohs micrographic surgery

## Abstract

Managing large nasal defects following Mohs surgery presents complex reconstructive challenges due to the nose's prominent and visible nature. These cases require a careful balance of preserving structural integrity, optimizing cosmetic outcomes, and maintaining vascular health. In situations where primary closure is impractical due to defect size and location, innovative techniques like the double rhomboid transposition flap offer versatile solutions, addressing both aesthetic concerns and functional requirements. The double rhomboid flap allows surgeons to achieve continuity of surrounding tissue, ensuring aesthetically pleasing texture, color, and thickness while minimizing complications like skin tension and potential airway issues. This case highlights the reconstructive challenges faced in managing large nasal defects following Mohs micrographic surgery for basal cell carcinoma. An 84-year-old male presented with a significant nasal defect following Mohs surgery that involved the dorsum, sidewall, tip, and ala, complicating primary closure due to skin tension and cosmetic concerns. Utilizing a double rhomboid transposition flap technique allowed for effective aesthetic and structural reconstruction, addressing skin tension and preserving nasal symmetry. This case emphasizes the importance of tailored reconstructive strategies to achieve optimal cosmetic and functional outcomes in complex nasal Mohs defects.

## Introduction

The rhomboid transposition flap is a sophisticated and versatile closure technique used in Mohs micrographic surgery, particularly for reconstructing defects resulting from the excision of non-melanoma skin cancers. This method is highly valued for its ability to achieve excellent aesthetic outcomes while preserving the natural architecture of the skin.

Mohs micrographic surgery involves the precise removal of skin cancer layer by layer, with each layer immediately examined under a microscope to ensure complete cancer removal while preserving as much healthy tissue as possible, allowing for clear margins to be achieved on the same day. In contrast, with classic surgical excision, patients often have to wait days for pathology results to determine if clear margins were obtained, potentially requiring additional surgery. However, Mohs surgery often leaves behind significant skin defects that require careful reconstruction. The rhomboid transposition flap addresses these challenges by utilizing local tissue to cover the defect, ensuring that skin texture, thickness, and color are carefully matched to create a more cohesive and natural appearance.

The technique involves creating a rhomboid-shaped flap adjacent to the defect, which is then transposed to cover the surgical site [1]. This approach ensures that the orientation of the flap and the resulting scar align with natural skin lines and creases, thereby enhancing both functional and cosmetic results. By utilizing this method, Mohs surgeons can achieve optimal closure with minimal donor site morbidity, leading to both effective reconstruction and aesthetically pleasing outcomes. A large defect of the nose poses a reconstructive challenge due to the prominence and visibility of the area. When dealing with lesions affecting the entire nasal unit, including the nasal sidewall, it's important to address both structural support and cosmetic outcomes. The reconstruction must avoid distortion of nasal symmetry, minimize tension in the surrounding area, and preserve airway function. Furthermore, careful hemostasis and preservation of vascular integrity must be prioritized in this richly vascularized area. The location, depth, and size of the defect determine the options available for reconstruction [2].

## Case presentation

**Figure 1:**
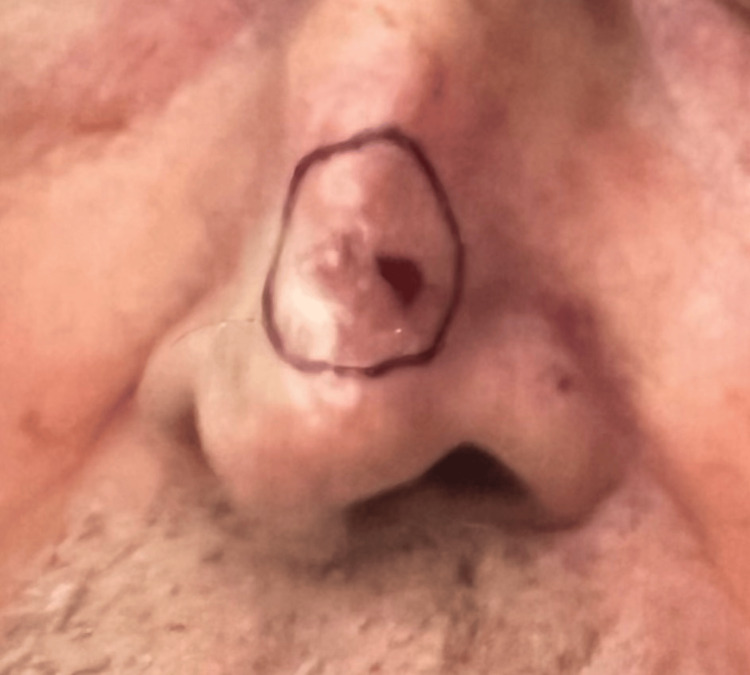
Pearly telangiectatic papule on the dorsal nose, confirmed as nodular basal cell carcinoma on histology

**Figure 2:**
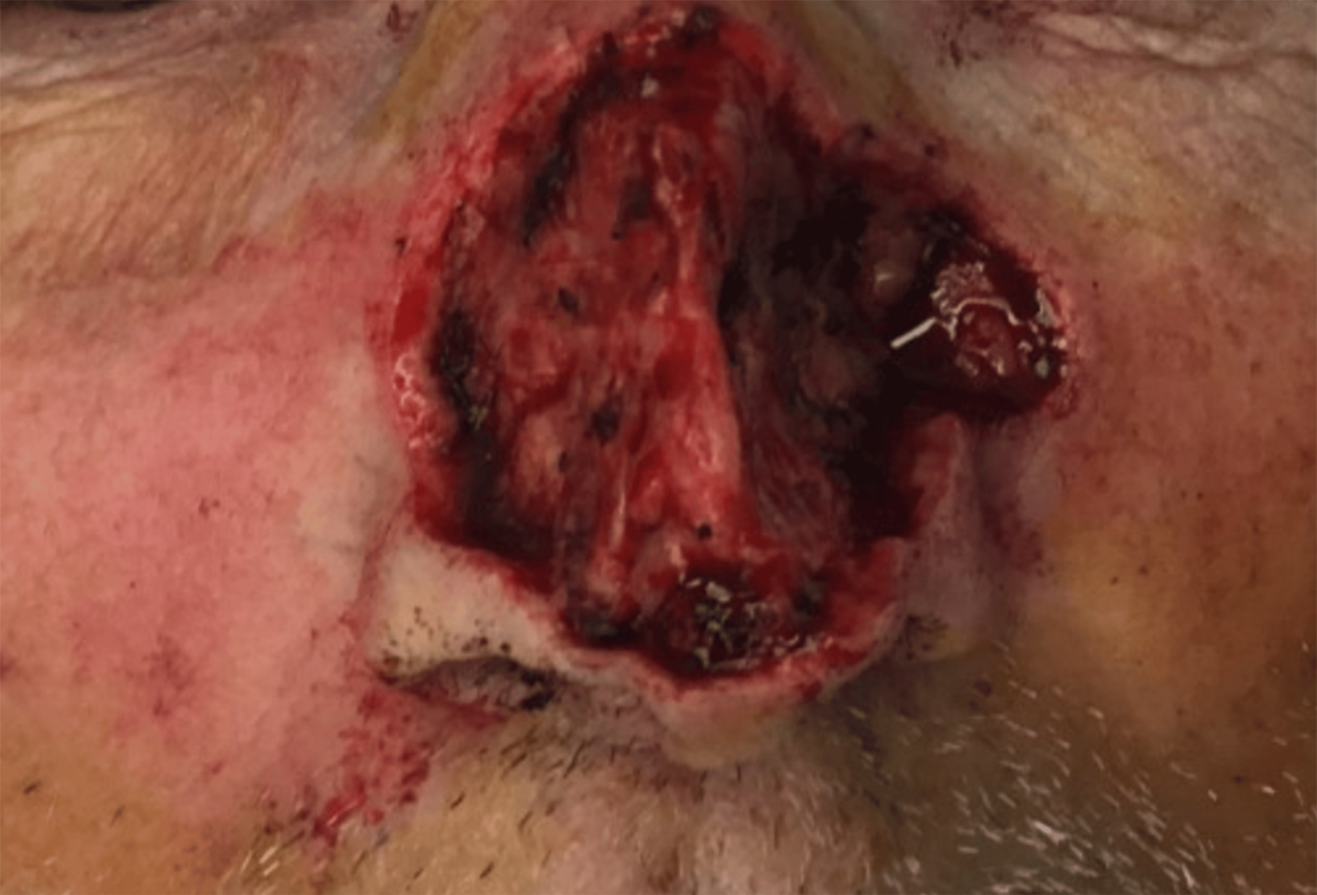
The 6 cm x 4 cm defect after six stages of Mohs micrographic surgery involving the nasal sidewall, dorsum, and tip

An 84-year-old Caucasian male with a history of non-melanoma skin cancer presented for Mohs micrographic surgery to remove a pearly telangiectatic papule on the dorsum of the nose (Figure 1), which histology confirmed as nodular basal cell carcinoma. The surgical procedure was performed in six stages and clear margins were achieved, leaving a 6 cm x 4 cm defect (Figure 2).

**Figure 3:**
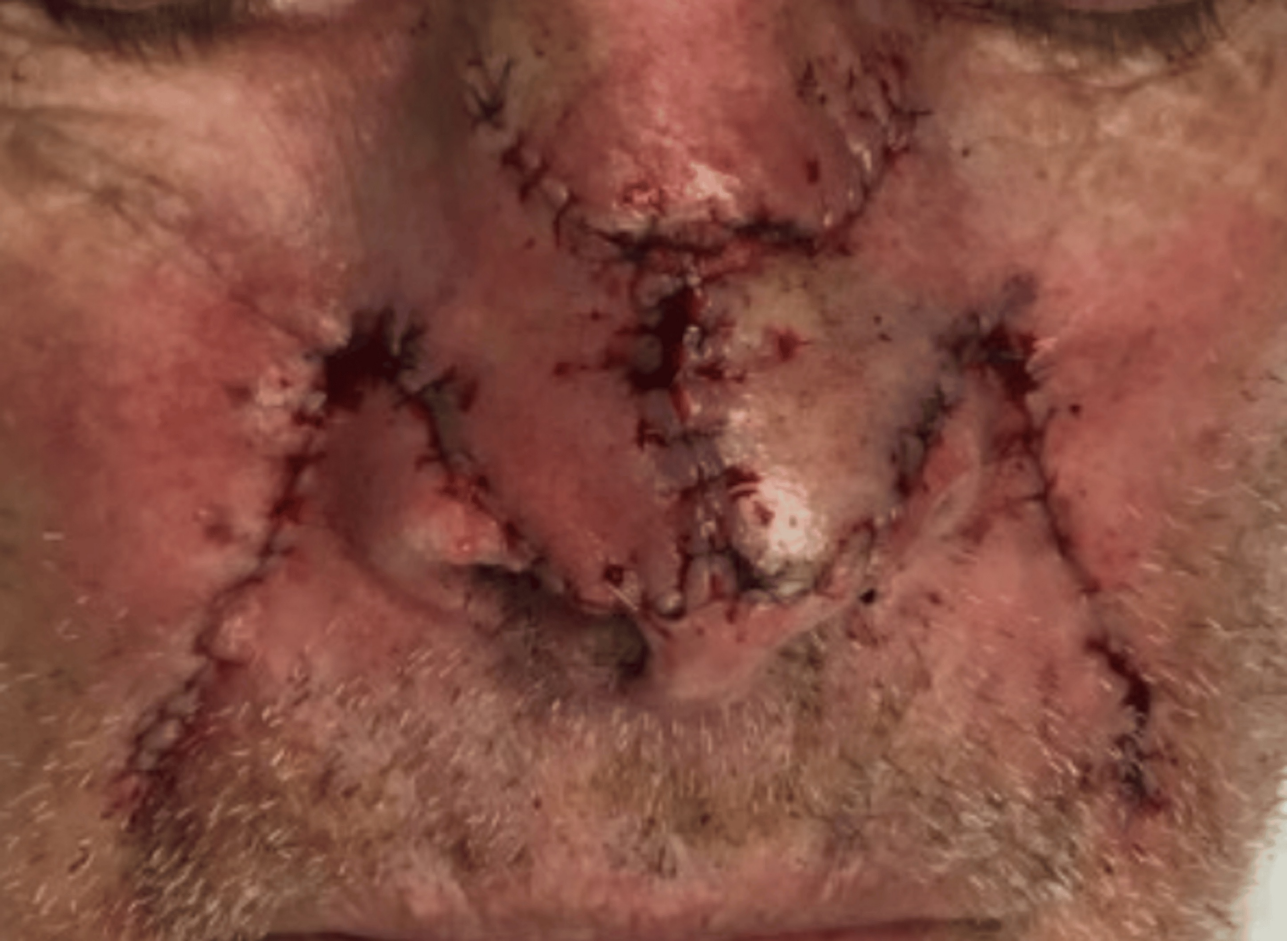
The post-reconstruction defect with a W-shaped scar, aligned with natural crease lines for improved concealment

The defect and surrounding area were locally anesthetized with 1% lidocaine with 1:100,000 epinephrine. The site was then cleansed with chlorhexidine solution and draped with a sterile drape. Secondary defects from the bilateral nasolabial folds were created using a 15-blade, and the skin was transposed onto the primary defect utilizing a double rhomboid transposition flap technique. Extensive undermining was performed to decrease wound tension. The wound was closed with 4-0 Biosyn as a subcuticular suture and with 5-0 Biosyn as a running suture to approximate the epidermal wound edges (Figure 3).

Tacking sutures were placed at the naso-facial sulcus to improve the patient's cosmetic appearance. Post-reconstruction, a gentle pressure dressing was placed on the surgical wound. The patient had an uneventful postoperative course. One week after the procedure, the patient returned for suture removal, having experienced no complications such as infection or nerve damage symptoms. The patient was subsequently lost to follow-up.

## Discussion

The Mohs defect, in this case, involved a large area of the patient’s nose, encompassing the nasal dorsum, sidewall, tip, and ala, with the depth extending to the septal cartilage. Given the size and location of the defect, a primary linear closure was not feasible due to the anticipated skin tension and potential for unfavorable cosmetic outcomes. One study's algorithm for closing post-Mohs nasal defects of ≥1.5 cm under local anesthesia rarely recommended using a transposition flap for defects exceeding 3 cm, except for nasal sidewall defects [3]. Although there is limited research specifically addressing the use of the double rhomboid transposition flap for large nasal defects, its demonstrated versatility and effectiveness in various dermatologic surgeries support its application in our case [4]. This technique’s ability to manage skin tension and maintain tissue continuity makes it valuable when conventional methods are less feasible. By utilizing tissue from opposite sides of the defect, the double rhomboid flap reduces directional tension, preserving anatomical landmarks and preventing distortion. The rhomboid-to-W technique further enhances the cosmetic outcome by converting the defect into a W-shaped scar that aligns with natural crease lines, resulting in better scar concealment and reduced postoperative scar contracture. Although forehead flaps, like the paramedian forehead flap, are commonly used for complex nasal reconstructions [3,5,6], they often require a multi-staged procedure. This approach was not ideal for our patient due to their preferences for treatment and recovery. The double rhomboid transposition flap offered a versatile and effective solution by redistributing skin tension and preserving airway function. It also maintained the continuity of the surrounding tissue in terms of texture, color, and thickness, and was completed in a single Mohs session.

## Conclusions

The double rhomboid transposition flap proved to be an effective technique for reconstructing a large, complex nasal defect in this case. By addressing skin tension and maintaining nasal symmetry, this method achieved both functional and cosmetic goals. The successful outcome highlights the double rhomboid flap as a valuable option for extensive nasal reconstructions where primary closure is impractical. This scenario demonstrates the double rhomboid transposition flap's effectiveness in restoring both function and cosmetic appearance, essential components in the holistic approach of Mohs micrographic surgery.
